# Discovery of Orphan Olfactory Receptor 6M1 as a New Anticancer Target in MCF-7 Cells by a Combination of Surface Plasmon Resonance-Based and Cell-Based Systems

**DOI:** 10.3390/s21103468

**Published:** 2021-05-16

**Authors:** Yae Rim Choi, Jaewon Shim, Jae-Ho Park, Young-Suk Kim, Min Jung Kim

**Affiliations:** 1 Research Group of Natural Materials and Metabolism, Korea Food Research Institute, Wanju 55365, Korea; uiu7895@naver.com (Y.R.C.); jwshim@kosin.ac.kr (J.S.); 2 Department of Food Science and Engineering, Ewha Womans University, Seoul 03760, Korea; yskim10@ewha.ac.kr; 3 Department of Biochemistry, Collage of Medicine, Kosin University, Busan 49267, Korea; 4 Research Group of Healthcare, Korea Food Research Institute, Wanju 55365, Korea; jaehopark@kfri.re.kr

**Keywords:** olfactory receptor, OR6M1, surface plasmon resonance, anthraquinone, MCF-7 breast cancer cell line, rutin

## Abstract

Olfactory receptors (ORs) account for 49% of all G protein-coupled receptors (GPCRs), which are important targets for drug discovery, and hence ORs may also be potential drug targets. Various ORs are expressed in breast cancer cells; however, most of them are orphan receptors, and thus, their functions are unknown. Herein, we present an experimental strategy using a surface plasmon resonance (SPR) system and a cell-based assay that allowed the identification of orphan OR6M1 as a new anticancer target in the MCF-7 breast cancer cell line. After the construction of stable OR6M1-expressing cells, the SPR-based screening of 108 chemicals for ligand activity was performed against OR6M1-expressing whole cells (primary screening) or membrane fragments (secondary screening). As a result, anthraquinone (AQ) and rutin were discovered to be new OR6M1 ligands. Based on calcium imaging in OR6M1-expressing Hana3A cells, AQ and rutin were classified as an OR6M1 agonist and antagonist, respectively. Cell viability and live/dead assays showed that AQ induced the death of MCF-7 cells, which was inhibited by rutin. Therefore, OR6M1 may be considered an anticancer target, and AQ may be considered a chemotherapeutic agent. This combined method can be widely used to discover the ligands and functions of other orphan GPCRs.

## 1. Introduction

G protein-coupled receptors (GPCRs), which are expressed in the cell membrane, are the most widely used targets of drugs, including anticancer agents, with approximately 36% of FDA-approved drug targets [1]. Among more than 800 GPCRs, only 36% have well-defined biological ligands, while another 15% are orphan GPCRs, and 49% are olfactory receptors (ORs) [2]. Orphan GPCRs are structurally similar to other GPCRs; however, their ligands are unknown. Most of the approximately 1000 and 390 OR genes in mice and humans, respectively, are orphan GPCRs [3], which is due to difficulties in protein expression in heterologous systems and the low stability of purified proteins [4]. Considering the high proportion of ORs in the GPCR superfamily, the identification of ligands for orphan ORs and investigation of their potential as anticancer targets are necessary.

Breast cancer is one of the most commonly diagnosed malignancies and the second leading cause of cancer-related death in women [5]. Owing to a variety of biological and genetic causes and various subtypes, breast cancer is treated with surgery, radiation therapy, chemotherapy, and hormone therapy [6]. Almost 80% of patients with breast cancer receive chemotherapy among other treatments; however, there is still no complete cure for breast cancer, and some side effects are always present [7]. Therefore, new chemotherapy targets and drugs are continually being developed, and ectopic ORs may have potential as new targets.

Human breast cancer tissues and cell lines highly express ORs, among which only three, OR2W3, OR2B6, and OR2T6, have established functions [8,9]. OR2W3 and OR2B6 were involved in breast cancer invasion and proliferation. OR2T6, which is overexpressed in breast cancer cell lines (MCF-7 and MDA-MB-231), was associated with the progression of breast cancer cells via the epithelial–mesenchymal transition (EMT)-mediated mitogen-activated protein kinase (MAPK) pathway and suppression of apoptosis. Since other orphan ORs expressed in breast cancer cells may have the potential to serve as pharmacological targets, ligands must be discovered for orphan ORs.

Surface plasmon resonance (SPR) allows for the direct detection of protein–ligand interactions and is, thus, a powerful tool for high-throughput screening (HTS) for novel ligands [10]. The reactivity of a ligand toward a target protein can be observed by immobilizing the target protein on an SPR chip and flowing the ligand over the chip in a mobile phase. The SPR technique benefits from being label free and from measures binding the kinetics and affinity of small molecules, peptides, and proteins to target proteins, such as GPCRs, and the technique requires only small amounts of proteins and chemicals for analysis [11,12].

Cells and microorganisms can be immobilized on an SPR chip to perform binding assays [12,13], which is very important for ligand discovery of orphan ORs by HTS. Generally, a purified membrane protein or a membrane protein in a membrane-mimicking structure is immobilized on an SPR chip and retains its native conformation [14,15]. However, it is difficult to isolate and purify ORs while maintaining their activity without structural modification. Therefore, for the discovery of ligands of orphan ORs, a primary screening should be performed, usually with low sensitivity, using orphan OR-expressing intact cells, and then a secondary screening should be performed using a method with improved sensitivity.

Herein, we present an experimental strategy that allowed the identification of human OR6M1, one of the orphan ORs expressed in MCF-7 cells, as a new anticancer target. New OR6M1 ligands were discovered using an SPR-based biosensor, and their effects on MCF-7 cells were evaluated. OR6M1 was selected based on its expression at the gene and protein levels in MCF-7 cells, as determined using polymerase chain reaction (PCR) and western blotting.

To discover OR6M1 ligands, primary and secondary screening of 108 chemicals was performed using OR6M1-expressing intact cells and membrane fragments, respectively, in an SPR-based binding assay. Finally, the function of OR6M1 in MCF-7 cells was investigated using the discovered ligands. This strategy can be used in the first phase of functional studies on orphan ORs and will be useful in confirming the potential of orphan GPCRs, including ORs, as drug targets.

## 2. Materials and Methods

### 2.1. Reagents

Anthraquinone (AQ), rutin, dimethyl sulfoxide (DMSO), sodium acetate, carbohydrazide, sodium cyanoborohydride, and amphotericin B were purchased from Sigma–Aldrich. Natural Product Libraries were provided by Selleck Chemicals. Dulbecco’s phosphate-buffered saline (DPBS) was from Welgene (Daegu, Korea), and HBS-EP buffer was from GE Healthcare Life Sciences (Chicago, IL, USA). 1-Ethyl-3-(3-dimethylaminopropyl)carbodiimide hydrochloride (EDC), N-hydroxysuccinimide (NHS), and ethanolamine were purchased from XanTec bioanalytics GmbH (Muenster, Germany).

### 2.2. Cell Culture

HEK293T/17 and MCF-7 cells were purchased from the American Type Culture Collection. HEK293T/17 cells were cultured in DMEM (Gibco, Grand Island, NY, USA) with 10% fetal bovine serum (FBS; Gibco) and 1% penicillin/streptomycin (P/S; Gibco). MCF-7 cells were cultured in RPMI 1640 medium (Gibco) with 10% FBS and 1% P/S. The Hana3A cells were a gift from Dr. Hiroaki Matsunami (Duke University Medical Center) and were cultured in DMEM containing 10% FBS, 1% P/S, and 6 μg/mL amphotericin B. All cells were cultured at 37 °C in an atmosphere of 5% CO_2_.

### 2.3. PCR and Western Blotting

For PCR, the total RNA was extracted from MCF-7 cells using the TRIzol reagent (Invitrogen, Carlsbad, CA, USA), and cDNA was synthesized using the SuperScript RT kit (Invitrogen). The cDNA was amplified with PCR using the following primer pairs: (1) *OR6M1* (forward: 5′-GTTTATCCTCTTGGCGGTGA-3′ and reverse: 5′-ACCTGAAGAAGAGGGGCAAT-3′) and (2) *GAPDH* (forward: 5′-CGAGATCCCTCCAAAATCAA-3′ and reverse: 5′-GGTGCTAAGCAGTTGGTGGT-3′). The amplified products were detected by agarose gel electrophoresis. *GAPDH* was used as a housekeeping gene.

For western blotting, the total proteins were extracted using RIPA buffer from the cells and cell membranes and were quantified using a bicinchoninic acid protein assay kit (Thermo Fisher Scientific, Waltham, MA). Equal amounts of protein were separated by sodium dodecyl sulfate polyacrylamide gel electrophoresis and transferred onto polyvinylidene difluoride membranes (Bio-Rad, Hercules, CA, USA). The membranes were blocked with 5% bovine serum albumin (BSA; Sigma, St. Louis, MO, USA) in Tris-buffered saline with 0.05% Tween 20 (TBST) for 1 h and then incubated with primary antibodies against OR6M1 (Invitrogen) and β-actin at 4 °C overnight. After being washed with TBST, the membranes were incubated with anti-mouse IgG secondary antibodies for 2 h. Chemiluminescence detection was carried out using the EZ-Western Lumi Pico ECL solution kit (DoGen, Seoul, Korea) and detected by Fusion solo S (Vilber Lourmat, Collégien, France).

### 2.4. Construction of a Stable OR6M1-Expressing Cell Line

For plasmid construction, the *OR6M1* gene was amplified by PCR (primers: 5′-ATCCTCTAGAGTCGACATGTCCCCTATACTAGGT-3′ and 5′-GGCCGCCCGGGTCGACTCAAGTTTTCCTTTGTAT-3′) using an *OR6M1* cDNA. The glutathione S-transferase gene (*GST*) was fused to the N-terminus of *OR6M1*, and the sequence was inserted into the pIRESpuro3 mammalian expression vector (Clontech Laboratories, Mountain View, CA, USA). The receptor-transporting protein 1 (*RTP1*) gene was amplified by PCR (primers: 5′-AAAGAATTCATGAGGATTTTTAGACCG-3′ and 5′-AAAGAATTCCTATACGGAGCTACGGAAAGA-3′) using *RTP1* cDNA. The amplified PCR product was inserted into the pIRESpuro3-GST-OR6M1 vector. The constructed plasmid is referred to as pIRES-RTP1-IRES-GST-OR6M1.

A stable OR6M1-expressing cell line was generated using a standard transfection method. HEK293T/17 cells were seeded in a six-well plate and transfected with pIRES-RTP1-IRES-GST-OR6M1 using Lipofectamine 3000 (Invitrogen) following the manufacturer’s instructions. Two days later, transfected cells were exposed to media containing 5 μg/mL puromycin. After selecting resistant colonies, single cells were seeded into each well of a 96-well plate. When single colonies reached high confluence, selected colonies were transferred to a 12-well plate. The successful generation of a stable OR6M1-expressing cell line was confirmed using immunofluorescence.

In brief, the cells were washed twice with DPBS and fixed with 4% paraformaldehyde for 10 min. The fixed cells were incubated with 5% BSA in DPBS for 1 h and then incubated with a GST antibody (Cell Signaling Technology, Beverly, MA, USA) at 4 °C overnight, followed by incubation with an Alexa Fluor 488-tagged anti-IgG secondary antibody (Invitrogen) for 1 h in the dark. After the cells were washed, the nuclei were stained with 4′,6-diamidino-2-phenylindole (Invitrogen) for 5 min. Finally, the coverslips were embedded in an antifade mounting solution and observed. The stable OR6M1-expressing cell line is referred to as OR6M1 cells.

### 2.5. Isolation of Membrane Fragments

Fragmentation of the cell membrane was performed using the MEM-PER Plus membrane protein extraction kit (Thermo Fisher Scientific) according to the manufacturer’s instructions. In brief, cells were harvested using a scraper and then centrifuged at 300× *g* for 5 min. The pellet was washed with the Cell Wash Solution, then resuspended in the Permeabilization Buffer, and incubated at 4 °C for 15 min. The permeabilized cells were centrifuged at 16,000× *g* for 15 min, then resuspended in the Solubilization Buffer and incubated at 4 °C for 30 min with constant mixing. Finally, the solubilized cells were centrifuged at 16,000× *g* for 15 min, and the supernatant containing solubilized membranes was collected.

### 2.6. Scanning Electron Microscopy (SEM) and Size Distribution

Cells and membrane fragments were observed under a field emission scanning electron microscope (FESEM, SUPRA 55VP, Jena, Germany). The size distributions of the cells and membrane fragments were based on dynamic light scattering (DLS) measurements using a Zetasizer Nano ZS instrument (Malvern Instruments, Ltd., Malvern, UK).

### 2.7. SPR

**Figure 1:**
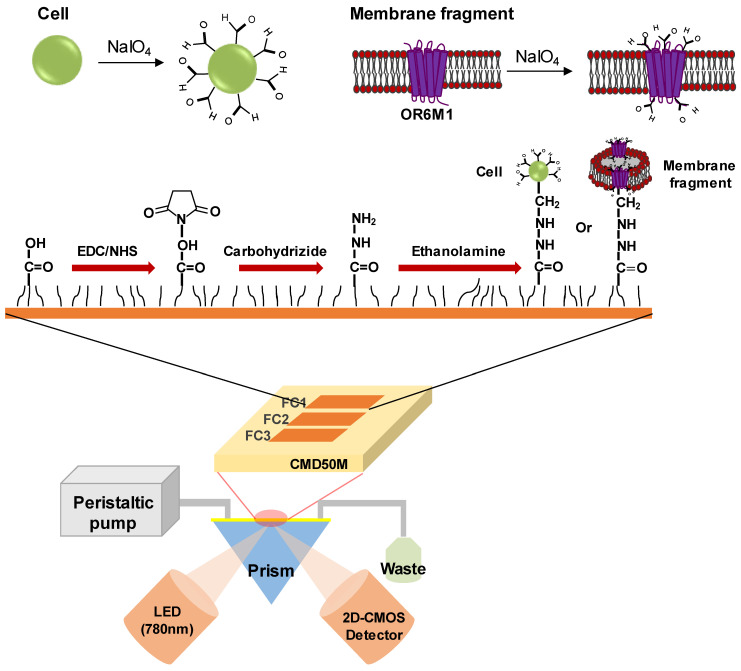
Schematic illustration of a surface plasmon resonance (SPR) biosensor with immobilized stable cells or membrane fragments expressing the olfactory receptor 6M1 (OR6M1). EDC, 1-Ethyl-3-(3-dimethylaminopropyl)carbodiimide hydrochloride; NHS, N-hydroxysuccinimide; CMD, carboxymethyl dextran; FC, flow channel; LED, light emitting diode; and 2D-CMOS, two dimensional-complementary metal oxide semiconductor.

The SPR experiments were conducted using an iMeasy300 SPR system (iCluebio, Seoul, Korea) with three flow channels (FC1-3), and the experimental process is shown in Figure 1.

#### 2.7.1. Cell and Membrane Fragment Oxidation

For the oxidation, cells were suspended at a density of 2 × 10^5^ cells/mL in 1 mL of 100 mM sodium acetate buffer (pH 5.5 (buffer A; Invitrogen), containing 1/50 volume of a fresh sodium metaperiodate solution), incubated on ice for 20 min, and centrifuged at 2000 rpm for 2 min. The pellet was washed twice with 10 mM sodium acetate buffer, pH 4.0 (buffer B). To obtain oxidized membrane fragments, the cells were suspended in buffer A, and membrane fragments were isolated as described above. The membrane fragments in the solubilization buffer were passed through a PD-10 desalting column (GE Healthcare) and eluted with buffer B.

#### 2.7.2. Immobilization of Oxidized Cells and Membrane Fragments on an SPR Sensor Chip

A carboxymethyl dextran sensor chip (CMD50M; XanTec bioanalytics, Germany), placed in the iMeasy300 system, was washed with HBS-EP buffer. The surface of the CMD50M chip was modified by sequentially treating the chip with a mixture of 0.1 M NHS and 0.4 M EDC (1:1), 5 mM carbohydrazide, and ethanolamine. Thereafter, the oxidized cells or membrane fragments were injected to be immobilized on the CMD50M chip. Finally, 0.1 mM sodium cyanoborohydride was injected to stabilize the immobilized cells or cell membrane fragments. The flow rate for all processes was 30 μL/min.

#### 2.7.3. Compound Binding Assay

Chemicals were diluted in HBS-EP buffer to the final concentrations shown in Table S1, which resulted in a final DMSO concentration of 0.1%. The 0.1% DMSO in HBS-EP buffer was used as a running buffer. The injection times for the baseline (buffer), chemicals, and dissociation were 5, 10, and 10 min, respectively. All assays were performed at 25 °C.

#### 2.7.4. Data Analysis

The amount of bound chemical designated as the change in the response unit (ΔRU) was calculated by subtracting the RU value for reference from that for the chemical solution. The ΔRU value between the HEK293T/17 cell/membrane fragment and reference was used as a negative control. The limit of detection (LOD) is defined by International Union of Pure and Applied Chemistry [16]. The LOD value is calculated as follows: ${\mathrm{LOD}=X}_{\mathrm{BL}}{+k\sigma }_{\mathrm{BL}}$. X_BL_ is the mean of the blank measurement without analyte, *k* is a numerical factor (typically 3 for LOD), and σ_BL_ is the standard deviation of blank measurement. All SPR experiments were analyzed using the iMeasy300 software.

### 2.8. Calcium Imaging

Hana3A cells transfected with pIRES-RTP1-IRES-GST-OR6M1 and MCF-7 cells were grown in a 96-well plate and incubated for 30 min with 10 μM Fura-2AM (Molecular Probes) in 4-(2-hydroxyethyl)-1-piperazineethanesulfonic acid (HEPES) buffer (10 mM HEPES, 2 mM CaCl_2_, 145 mM NaCl, 10 mM D-glucose, 5 mM KCl, and 1 mM MgSO_4_). Calcium images were obtained using a Zeiss inverted microscope coupled with the DG4 system (Sutter, Inc.) and displayed with the Metafluor software. The Fura-2 ratiometric fluorescence (F_340_/F_380_) indicated relative changes in the intracellular calcium concentrations ([Ca^2+^]_i_).

### 2.9. Cell Viability Assay

MCF-7 cells were incubated in a 96-well plate for 24 h and then treated with AQ (0, 25, 50, and 100 μM), rutin (0, 25, 50, and 100 μM), or AQ (0, 25, 50, and 100 μM) + rutin (100 μM) for 48 h. Then, the cell viability was evaluated by incubating the plates with the Cell Counting Kit-8 (CCK-8) solution for 2 h and measuring the absorbance at 450 nm, with the reference absorbance at 650 nm, as follows: Cell viability (%) = (Abs_sample_ − Abs_ref_)/Abs_control_ × 100. For the live/dead fluorescence assay, the cells were stained using the LIVE/DEAD^®^ cell imaging kit (Invitrogen) and visualized using a fluorescence microscope (Axio Observer A1; Carl Zeiss AG, Jena, Germany). The images of live and dead cells were green and red, respectively.

### 2.10. Statistical Analysis

All experiments were performed in triplicate, and statistical analysis was performed using the GraphPad Prism 5 software, presented as the mean ± standard deviation. One-way ANOVA was used for comparisons between groups, followed by Dunnett’s post-test. Values of 0.05(*) and 0.001 (***) were considered statistically significant.

## 3. Results and Discussion

### 3.1. OR6M1 Expression in MCF-7 Cells and the Construction of OR6M1 Cells

**Figure 2:**
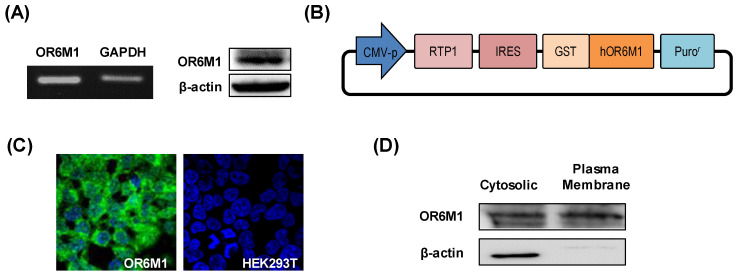
Characterization of OR6M1 expression. (**A**) Detection of OR6M1 expression in MCF-7 cells by reverse transcription–polymerase chain reaction (left) and western blotting (right). (**B**) Plasmid construct containing the receptor-transporting protein 1 (*RTP1*) gene, glutathione S-transferase (GST)-tag, and human OR6M1 gene. (**C**) Immunocytochemical detection of OR6M1 in the constructed OR6M1 cell line. GST-tagged OR6M1, green; nuclei, blue. Live cells, green; dead cells, red; (**D**) OR6M1 expression in cells and membrane fragments.

Gene and protein expression of OR6M1 in MCF-7 cells was first confirmed by reverse transcription-PCR and western blotting, respectively (Figure 2A). To the best of our knowledge, this is the first report of the detection of specific bands of OR6M1 in MCF-7 cells, although the expression and function of some ORs have been previously reported in breast cancer.

The analysis of 960 breast tumors and 56 breast cancer cell lines showed the abundant expression of 21 OR genes, and, in particular, OR2W3 and OR2B6 were highly associated with breast cancer invasion and proliferation, respectively [8]. In addition, OR2T6 expression in two breast cancer cell lines, MCF-7 and MDA-MB-231, promoted cell proliferation, migration, and invasion via the EMT-mediated MAPK pathway and suppressed cell apoptosis [9]. However, the function of some ORs, including OR6M1, has not yet been confirmed in MCF-7 cells.

To establish the role of OR6M1 in MCF-7 cells by identifying OR6M1 ligands, OR6M1 cells were generated by transfecting HEK293T/17 cells with the recombinant plasmid pIRES-RTP1-IRES-GST-OR6M1. The latter was constructed by inserting the target *OR6M1* gene, the *RTP1* gene as a chaperone, a GST-tag, and pIRES-puro into a eukaryotic expression plasmid (Figure 2B). The successful generation of OR6M1 cells was confirmed by detecting GST-tagged OR6M1 (green fluorescence) in the cell membrane using immunofluorescence and by western blotting with an anti-GST antibody (Figure 2C,D).

No green fluorescence was observed in non-transfected HEK293T/17 cells. In general, ORs are poorly expressed on the surface of heterologous cells. Unlike other GPCRs, ORs require specific chaperone proteins, such as RTP1, RTP2, and REEP1, to facilitate successful surface expression [17]. A shorter form of RTP1, RTP1s, was shown to help express ORs in further studies [18,19]. Most studies used Hana3A cells, which stably express RTP1, RTP2, or REEP1, for transient OR expression; however, the reaction to compounds in transiently expressing cells depends on intracellular changes via OR signaling, not on direct binding. Meanwhile, our OR6M1 cells were more suitable for primary screening using a biosensor to observe the direct binding of compounds.

### 3.2. OR6M1 Cell-Based SPR Biosensor for Primary Screening

**Figure 3:**
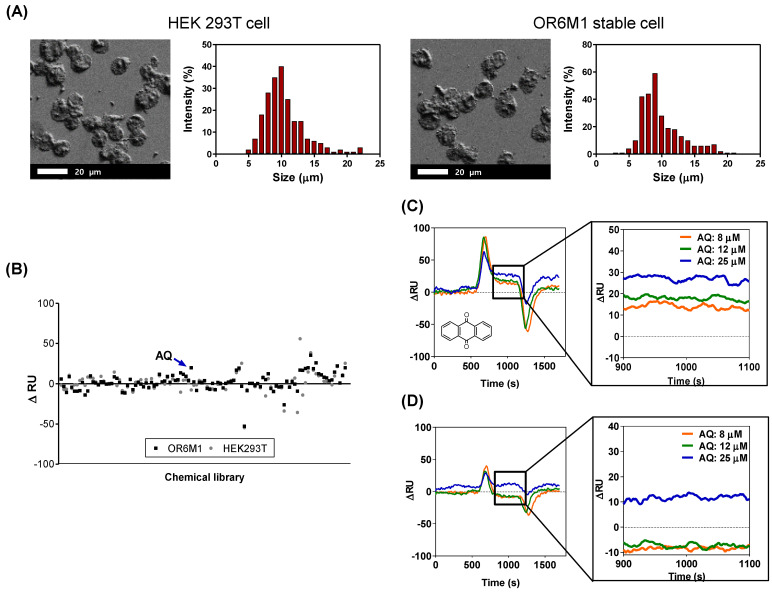
Identification of anthraquinone (AQ) as a putative OR6M1 ligand using a cell-immobilized SPR biosensor. (**A**) Scanning electron microscopy (SEM) images and size distributions of cells immobilized on a CMD50M sensor chip. (**B**) Cell-based screening of 108 chemicals for ligand activity. (**C**,**D**) Sensorgrams of AQ at three different concentrations (8, 12, and 25 μM) using CMD50M-chip-immobilized OR6M1 cells (**C**) or HEK293T/17 cells (**D**). The mean signal values between 900 and 1100 s were used as the detected signal value.

The SPR response to each of 108 compounds was monitored using the manufactured cell-based SPR biosensor. OR6M1 cells were immobilized by covalent bonding between the aldehyde group on the cell surface and carbohydrazide on the surface of the SPR chip. Immobilized cells were characterized using SEM (Figure 3A). The morphologies of the two cell lines, OR6M1 cells and HEK293T/17 cells, remained spherical, and the average cell diameters were 9 ± 1 μm.

The SPR response of each of the 108 compounds showed that only one chemical, AQ, was bound to OR6M1 cells (Figure 3B, Table S1). The ΔRU value of AQ at 15 μM was 20 RU in OR6M1 cells, which was higher than that in HEK293T/17 cells. The reactivity of AQ toward OR6M1 cells increased in a concentration-dependent manner (Figure 3C). The ΔRU values were measured as averages between 900 and 1100 s and were found to be 13, 19, and 28 RU at AQ concentrations of 8, 12, and 25 μM, respectively, using OR6M1 cells.

Given that the LOD value was 5.28 RU using OR6M1 cells, the responses at all AQ concentrations could be considered as a signal. As shown in Figure 3D, HEK293T/17 cells were responsive to AQ only at 25 μM. Although nonspecific binding was observed in AQ-treated HEK293T/17 cells at 25 μM, the ΔRU value was only 11.67 RU, which was less than that detected using OR6M1 cells (28 RU). Therefore, AQ could be considered an OR6M1 ligand in the primary screening.

The size of the material to be immobilized on a chip is one of the factors to be considered in SPR. The sensitivity of SPR detection increases as the distance to the detection surface decreases and is the best at a distance of 50 nm from the surface [20]. The evanescent field detectable with the SPR chip is up to 300 nm, and therefore, the binding reaction between a ligand and a receptor has to be within 300 nm [21]. However, the cells used in this study were 9 ± 1 μm, which was far beyond the detection range.

Nevertheless, one OR6M1 ligand was discovered, likely because (i) the binding reaction occurred in a portion of immobilized cells close to the chip and (ii) the compound–receptor interaction activated the intracellular signaling cascade, causing an SPR signal. As shown in Figure 3A, the density of the cells attached to the SPR chip was not high, which allowed the flowing chemicals to circulate around the cells and to be attached to the OR6M1 expressed on the cell membrane close to the chip surface. Several studies have examined the SPR response by immobilizing cells on SPR chips.

The RU values of RBL-2H3 and PAM212 cells on SPR chips were increased by binding an IgE antibody and EGF, respectively [22]. ORI7-expressing cells were activated on an SPR chip by various odorants, especially octanal, which increased the SPR signals [23]. SPR responses can also be generated by intracellular signal transduction, as chemical–OR interactions in the cell membrane activate intracellular signaling cascades, open ion channels, such as the cyclic nucleotide-gated (CNG) channel, and induce an influx of ions, such as Ca^2+^ [24].

Similarly, the release of histamine from human basophils via the IgE-induced activation of an intracellular signaling cascade was confirmed in real time via SPR [25]. Although cells can be used to detect ligands via SPR techniques, problems, such as nonspecific reactions, increased noise, and reduced sensitivity, can occur. To overcome these problems, the size of the material immobilized on an SPR chip needs to be reduced to allow the identification of additional ligands.

### 3.3. OR6M1 Cell Membrane Fragment Based SPR Biosensor for Secondary Screening

**Figure 4:**
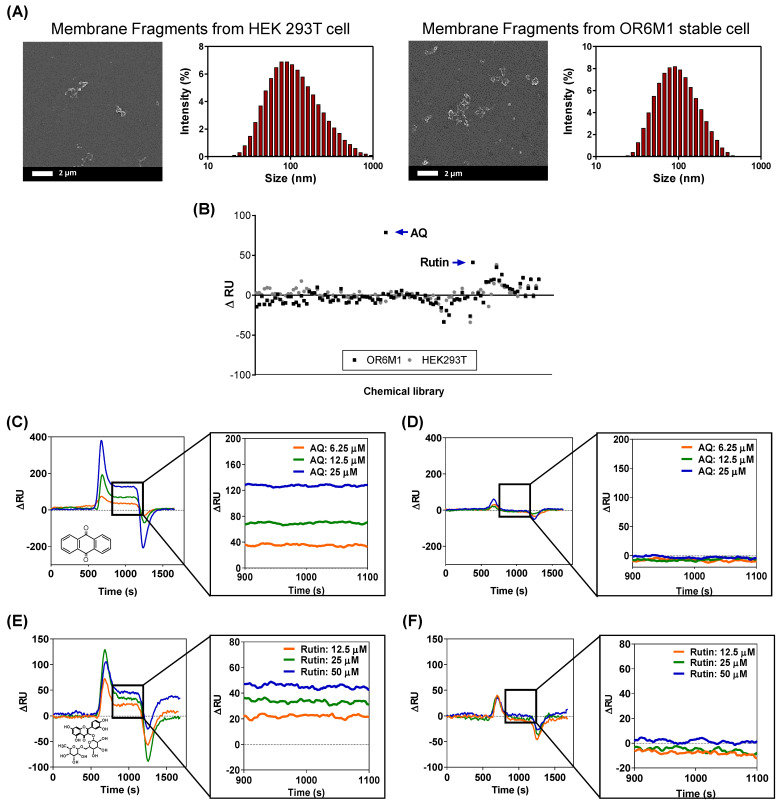
Identification of AQ and rutin as OR6M1 ligands using membrane fragment-immobilized SPR biosensor. (**A**) SEM images and size distributions of membrane fragments immobilized on a CMD50M sensor chip. (**B**) Membrane-fragment-based screening of 108 chemicals for ligand activity. (**C**,**D**) Sensorgrams of AQ at three different concentrations (6.25, 12.5, and 25 μM) using CMD50M-chip-immobilized OR6M1-expressing cell membranes (**C**) or HEK293T/17 cell membranes (**D**). (**E**,**F**) Sensorgrams of rutin at three different concentrations (12.5, 25, and 50 μM) using CMD50M-chip-immobilized OR6M1-expressing cell membranes (**E**) or HEK293T/17 cell membranes (F).

The smaller-sized cell membrane fragments were characterized by SEM and DLS (Figure 4A). The cells were effectively destroyed, and the average size of the membrane fragments was 124.78 ± 12.8 nm. In the secondary screening of the 108 chemicals using a membrane-fragment-immobilized SPR chip, AQ (15 μM) and rutin (166 μM) increased the ΔRU values in membrane fragments from OR6M1 cells compared with those in the control membrane fragments from HEK293T/17 cells (Figure 4B, Table S1).

The SPR responses to AQ were concentration dependent, with ΔRU values of 35, 70, and 130 RU at AQ concentrations of 6.25, 12.5, and 25 μM, respectively, in an OR6M1-expressing membrane-fragment-immobilized SPR chip (Figure 4C). However, no SPR signal was detected in the HEK293T/17 membrane-fragment-immobilized SPR chip (Figure 4D). The SPR responses to rutin were also concentration dependent, and the reactivity of rutin toward OR6M1-expressing membrane fragments was estimated to be 20, 30, and 49 RU at 12.5, 25, and 50 μM, respectively (Figure 4E). Meanwhile, no reactivity of rutin toward membrane fragments of HEK293T/17 cells was detected (Figure 4F).

The use of OR6M1-expressing membrane fragments not only effectively reduced the size and nonspecific binding compared with cells but also led to maintaining OR6M1 stability and improving the assay sensitivity. The average membrane fragment was 124 nm, i.e., approximately 72-times smaller than the cell, and nonspecific AQ binding at 25 μM, which was observed in HEK293T/17 cells, was not detected in HEK293T/17 cell membrane fragments. The stability of the OR6M1 structure in the membrane fragments was verified by the response to AQ in SPR.

AQ at 25 μM was successfully bound to OR6M1-expressing membrane fragments. The sensitivity improved as the size of the immobilized material decreased from intact cells to membrane fragments, which were within the SPR detection range (<300 nm). At the AQ concentration of 25 μM, the SPR signal detected in the membrane fragments (130 RU) was 4.64-times higher than that generated by the cells (28 RU). Moreover, the increase in sensitivity led to the discovery of an additional ligand. Using the membrane-fragment-based SPR biosensor, rutin was identified as a new OR6M1 ligand, whereas it was not identified using the cell-based SPR biosensor.

GPCRs are the most widely used drug targets, and the most advanced SPR application is possible when they are in a purified form. However, the poor structural stability of purified GPCRs is a significant barrier to screening for new ligands and to understanding the structure and function of GPCRs. Therefore, artificial membrane-like environments, such as micelles, lipid vesicles, nanodiscs, and planar lipid membranes were developed for the proper functioning of GPCRs [26,27].

A membrane-mimicking environment was prepared by combining a membrane protein and lipid, and this was used to analyze and elucidate the function and structure of GPCRs, as well as to study protein–protein and protein–chemical interactions. However, membrane proteins used in artificial membrane-like environments are typically obtained from cell-free expression systems, which requires additional processing, including the denaturation and refolding of proteins and confirmation of their structure [28,29]. The similarity of a protein structure to that of a native protein can be inferred by examining the reactivity toward a ligand, which is impossible for orphan GPCRs.

Therefore, the best environment for biophysical studies of orphan GPCRs is a natural, non-artificial lipid bilayer. In this study, we were able to effectively obtain cell membrane fragments using a simple method. The membrane fragments not only demonstrated structural stability via the reactivity with AQ but also contributed to the discovery of new ligands. Cell- and membrane-fragment-based screening approaches are indispensable in the study of orphan GPCRs, and this study effectively demonstrated the potential of these experimental methods.

### 3.4. Classification of AQ and Rutin as an OR6M1 Agonist and Antagonist

**Figure 5:**
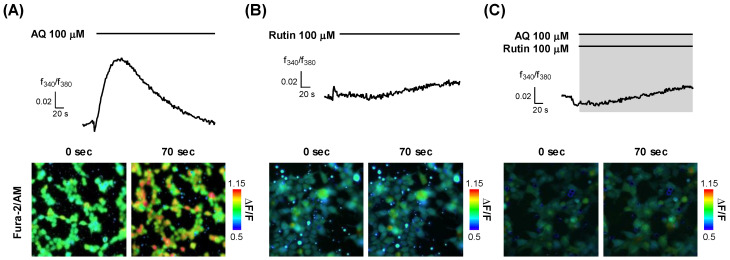
Modulation of [Ca^2+^]_i_ by AQ and rutin in OR6M1-transfected Hana3A cells. (**A**) AQ treatment; (**B**) rutin treatment, and (**C**) co-treatment with AQ and rutin. Time response recording of the Fura-2AM fluorescence ratios (340/380 nm) (upper panel) and fluorescent images (lower panel).

To elucidate the function of OR6M1 in MCF-7 cells, it was necessary to determine whether the AQ and rutin ligands were OR6M1 agonists or antagonists. Calcium imaging using OR6M1-transfected Hana3A cells showed that the Ca^2+^ signals increased in AQ-treated but not in rutin-treated cells (Figure 5A,B). The co-treatment of AQ and rutin completely blocked the AQ-induced Ca^2+^ signals in OR6M1-transfected Hana3A cells (Figure 5C). These data indicate that AQ was an OR6M1 agonist and rutin was an OR6M1 antagonist.

In OR cells, olfactory signal transduction is initiated by the binding of odorants to the OR, which, in turn, triggers an increase in cAMP levels and the opening of CNG channels, which allows the influx of cations—primarily sodium and calcium ions [30]. This transient increase in [Ca^2+^]_i_ triggers the opening of Ca^2+^-activated chloride channels, which amplify the CNG channel signal [31].

On the other hand, in Hana3A cells, the activation of an OR stimulates the secretion of Ca^2+^ from the endoplasmic reticulum into the cytoplasm, increasing the [Ca^2+^]_i_. Thus, an agonist increases the [Ca^2+^]_i_, while an antagonist does not change the [Ca^2+^]_i_ but inhibits the Ca^2+^ increase by the agonist. Based on the changes of [Ca^2+^]_i_ by AQ, rutin, and AQ + rutin, we concluded that AQ was an OR6M1 agonist and rutin was an OR6M1 antagonist.

### 3.5. Effects of AQ and Rutin in MCF-7 Cell

**Figure 6:**
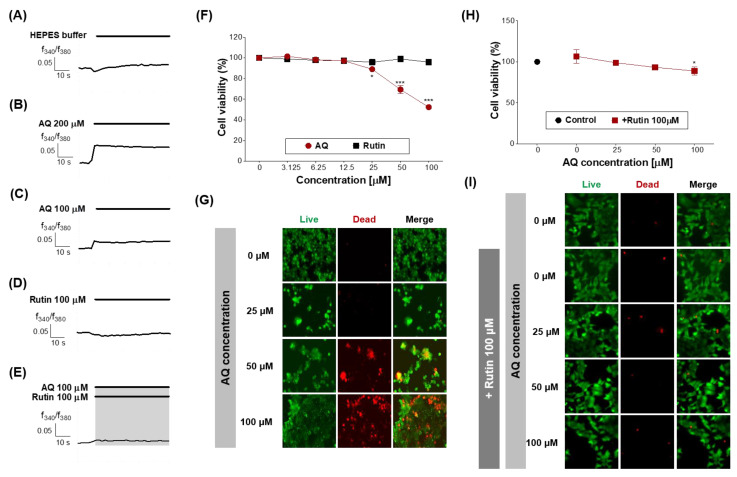
Effects of AQ and rutin on the breast cancer cell line, MCF-7. (**A**–**E**) Time response recording of [Ca^2+^]_i_ in Fura-2AM-loaded MCF-7 cells after treatment with 4-(2-hydroxyethyl)-1-piperazineethanesulfonic acid (HEPES), AQ (100 and 200 μM), rutin (100 μM), and AQ + rutin. (**F**) Concentration-response curves of the MCF-7 cell viability (%) after treatment with AQ or rutin for 48 h. (**G**) Live/dead cell imaging of MCF-7 cells treated with AQ (25–100 μM) for 48 h. Live cells, green; dead cells, red. (**H**) Concentration-response curves of the MCF-7 cell viability (%) after co-treatment with different concentrations of AQ and 100 μM rutin for 48 h. (I) Live/dead cell imaging of MCF-7 cells treated with AQ + rutin for 48 h. The data are expressed as the mean ± standard deviation. * *p* < 0.05 and *** *p* < 0.001 untreated vs. ligand-treated cells (one-way analysis of variance and Dunnett’s post-test).

Finally, the function of OR6M1 in MCF-7 cells was investigated using AQ and rutin. The calcium signal in MCF-7 cells was increased by AQ (100 and 200 μM) but was not changed by rutin (100 μM) and HEPES buffer (Figure 6A–D). The co-treatment of MCF-7 cells with AQ (100 μM) and rutin (100 μM) completely blocked the AQ-induced increase in the calcium signal (Figure 6E). Therefore, the response to AQ is likely to be initiated in MCF-7 cells by OR6M1.

Next, the effects of AQ and rutin on the MCF-7 cell viability were evaluated using CCK-8 and live/dead assays after 48 h of treatment. AQ significantly inhibited the viability of MCF-7 cells in a concentration-dependent manner compared to the control (Figure 6F), and caused MCF-7 cell death at the concentration of 50 and 100 μM (Figure 6G). AQ belongs to the quinone family, and AQ derivatives (AQs) naturally occur in plants, including *Heterophyllaea pustulata* Hook f. (Rubiaceae), senna leaves and pods, and aloe [32,33].

AQs have various biological functions, including antimicrobial, antifungal, antiplatelet, diuretic, and anticancer activities [34,35,36,37,38,39,40,41]. Some AQs have been reported to show effects on breast cancer cell lines. 1,3-Dihydroxy-9,10-anthraquinone-2-carboxylic acid induced G2/M cell cycle arrest and apoptosis in MCF-7 cells. Rubiadin and soranjidiol exhibited significant photocytotoxicity against MCF-7c3 cells [32,42]. However, the effects of AQ on MCF-7 cells and the target protein of AQ had not been previously reported. Our results revealed, for the first time, that the target protein of AQ in MCF-7 cells was OR6M1, and the activation of OR6M1 caused cell death.

Rutin treatment induced no change in the MCF-7 cell viability (Figure 6F), whereas the treatment with AQ in the presence of rutin (100 μM) effectively attenuated the inhibition of MCF-7 cell viability and the induction of MCF-7 cell death by AQ (Figure 6H,I). Rutin has several pharmacological effects, including cytoprotective, neuroprotective, anticancer, antioxidant, and vasoprotective activities [43,44,45,46,47]. Rutin showed anticancer efficacy against certain breast cancer cell lines [48].

Rutin treatment induced cytotoxicity, anticancer activity, cell cycle arrest at the G2/M and G0/G1 phases, and apoptosis in the breast cancer cell line MDA-MB-231 but had no effect on MCF-7 cells. Our finding was similar to a previous study but demonstrated for the first time that rutin suppressed the efficacy of an anticancer agent by binding to OR6M1 in MCF-7 cells.

AQ activated OR6M1 and, thereby, triggered MCF-7 cell death, whereas rutin suppressed AQ-induced cell death. Altogether, the results suggest that OR6M1 is involved in the viability and death of MCF-7 cells.

## 4. Conclusions

In this study, we presented an optimized strategy that shows the potential of orphan OR6M1 as a new drug target in the MCF-7 breast cancer cell line by integrating SPR-based ligand screening and cell-based assays. The SPR biosensors with immobilized whole cells and membrane fragments expressing OR6M1 were developed for primary and secondary ligand screening, respectively. The constructed OR6M1 cells stably expressed the OR6M1 protein in the cell membrane, and the OR6M1 protein presented in the membrane fragment was structurally stable by examining the reactivity to the ligand found in the OR6M1 cell-immobilized SPR biosensor.

In the ligand screening, the SPR biosensor with immobilized membrane fragments showed a higher sensitivity and selectivity compared with the immobilized whole cells. Based on the results of the SPR and calcium influx, the two identified OR6M1 ligands, AQ and rutin, were classified as an agonist and antagonist, respectively. Finally, this study demonstrated, for the first time, that the orphan OR6M1 was involved in the death of MCF-7 cells by examining the effects of AQ and rutin on MCF-7 cells.

AQ can be considered one of the candidates for anti-breast cancer drugs; however, further studies are needed, including research regarding the mechanism of AQ-induced MCF-7cell death and the effects of AQ on the migration and invasion of MCF-7 cells. This study provides a systematic method for drug discovery design by presenting the entire process of discovering the ligands of orphan GPCRs, which is difficult to express and difficult to maintain structural stability, discovering their efficacy in cancer cells, and confirming their potential as drug targets.

## Data Availability

Not applicable.

## Supplementary Materials

The following are available online at https://www.mdpi.com/article/10.3390/s21103468/s1, Table S1: Supplementary table.
