# Supplementary material for: Discovery of Orphan Olfactory Receptor 6M1 as a New Anticancer Target in MCF-7 Cells by a Combination of Surface Plasmon Resonance-Based and Cell-Based Systems
Source: Sensors (Basel). 2021 May 16;21(10):3468. doi: 10.3390/s21103468 (PMC8156394; doi:10.3390/s21103468)
Supplement: Supplementary file 1 [file sensors-21-03468-s001.zip › sensors-1218662-supplementary.pdf]

Article

Discovery of Orphan Olfactory Receptor 6M1 as a New Anticancer Target in MCF-7 Cells by a Combination of Surface Plasmon Resonance-Based and Cell-Based Systems

Yae Rim Choi [1,2], Jaewon Shim [1,3], Jae-Ho Park [4], Young-Suk Kim [2] and Min Jung Kim [1,*]

[1] Research group of Natural Materials and Metabolism, Korea Food Research Institute, Wanju 55365, Korea; uiu7895@naver.com (Y.R.C.); jwshim@kosin.ac.kr (J.S.)
[2] Department of Food Science and Engineering, Ewha Womans University, Seoul 03760, Korea; yskim10@ewha.ac.kr (Y.-S.K.)
[3] Department of Biochemistry, Collage of Medicine, Kosin University, Busan 49267, Korea
[4] Research Group of Healthcare, Korea Food Research Institute, Wanju 55365, Korea; jaehopark@kfri.re.kr
* Correspondence: mjkim14@kfri.re.kr; Tel.: +82-63-219-9380

Citation: Choi, Y.R.; Shim, J.; Park, J.-H.; Kim, Y.-S.; Kim, M.J.; Discovery of Orphan Olfactory Receptor 6M1 as a New Anticancer Target in MCF-7 Cells by a Combination of Surface Plasmon Resonance-Based and Cell-Based Systems. *Sensors* **2021**, *21*, 3468. https://doi.org/10.3390/10.3390/s21103468

Academic Editor: Raffaele Velotta
Received: 27 April 2021
Accepted: 12 May 2021
Published: 16 May 2021

Publisher's Note: MDPI stays neutral with regard to jurisdictional claims in published maps and institutional affiliations.

Copyright: © 2021 by the authors. Licensee MDPI, Basel, Switzerland. This article is an open access article distributed under the terms and conditions of the Creative Commons Attribution (CC BY) license (http://creativecommons.org/licenses/by/4.0/).

Table S1. Primary and secondary screening of 108 chemicals using SPR chip immobilized cells or membrane fragments expressing OR6M1.

No.	Natural products	Concentration (μM)	Resonance unit (RU)			
			HEK293T cells	OR6M1 cells	HEK293T membrane fragment	OR6M1 membrane fragment
1	Rapamycin	22	−1.70	5.99	2.97	−14.36
2	Artemisinin	202	−7.17	−3.38	3.23	−11.52
3	Lactulose	199	−6.79	−10.78	0.69	−1.16
4	Aminophylline	126	10.63	8.98	8.12	−11.41
5	Chenodeoxycholic Acid	201	−5.91	−4.20	9.60	−6.37
6	Capsaicin	200	−1.23	−9.09	9.05	−11.61
7	Baicalin	157	−1.23	−9.09	1.63	−7.45
8	Cinchonine	200	4.12	−8.63	2.00	−3.33
9	Docetaxel	201	5.89	1.50	−3.49	1.44
10	Celastrol	200	−1.94	−13.94	1.42	−5.30
11	Natamycin	11	8.38	−8.88	−8.48	−10.54
12	Paclitaxel	200	7.69	9.85	−0.32	−9.44
13	Streptozotocin	200	6.76	2.32	1.81	−1.58
14	Reserpine	21	2.21	1.00	1.81	−1.58
15	Hydrocortisone	201	−0.44	−1.19	7.00	−10.59
16	Daidzein	201	9.24	2.60	9.17	−8.92
17	3-Indolebutyric acid	202	3.37	2.20	3.18	−12.71
18	Arbutin	202	−0.38	−0.29	17.86	−0.57
19	Berberine chloride	108	0.36	−0.20	4.82	−6.84
20	Cryptotanshinone	17	0.57	0.34	8.56	−10.62
21	Doxorubicin HCl	172	14.56	8.56	6.73	4.48
22	Genistein	200	−2.31	6.89	2.57	2.69
23	Ursodiol	201	4.91	1.36	1.61	2.99
24	Estradiol	198	−8.46	−10.96	7.72	10.02

25	Vitamin B12	55	−10.44	−11.35	−0.13	−3.57
26	Lovastatin	20	−3.71	−2.01	−1.61	−6.01
27	4-Methylumbelliferon	199	5.15	5.57	0.90	0.42
28	Artesunate	200	−10.38	−7.43	3.90	−2.47
29	Caffeic Acid	200	1.76	−1.80	−6.54	−8.26
30	Cytisine	200	2.24	1.92	0.06	−6.33
31	2-Methoxyestradiol	199	5.07	−0.48	−0.84	−1.47
32	Ginkgolide B	200	−3.50	−3.75	−5.65	−6.26
33	Adenosine	45	5.34	1.00	4.32	8.28
34	Nicotinic Acid	203	−4.16	−4.87	−0.73	−1.45
35	Pregnenolone	70	6.49	1.73	−1.79	−9.49
36	Dextrose	200	−5.43	−2.86	−3.25	−8.64
37	Esculin	200	−1.39	−0.79	−0.53	−3.25
38	Asiatic Acid	198	9.90	11.90	1.02	−1.75
39	Chlorogenic Acid	200	1.45	2.22	−1.42	−9.26
40	Daidzin	199	−0.34	3.48	4.59	−5.18
41	Vincristine sulfate	108	7.69	9.19	−0.18	−1.43
42	Isotretinoin	200	−0.13	1.94	−1.42	−1.79
43	Tretinoin	200	5.95	10.45	−1.15	−1.98
44	Oxytetracycline	200	4.14	4.14	−1.34	−3.30
45	Methoxsalen	199	3.68	4.97	−4.60	−5.95
46	Xylose	200	−10.40	13.64	1.27	−9.76
47	Aloe-emodin	11	4.43	11.89	−2.53	−5.09
48	Azomycin	195	4.91	9.42	−9.29	−5.55
49	Chrysin	201	1.27	4.24	2.49	1.71
50	Anthraquinone	15	−7.69	20.00	−7.52	78.85
51	Dihydroartemisinin (DHA)	197	−1.80	2.39	0.18	1.20
52	Acarbose	155	−1.26	−8.37	4.63	−2.17
53	Resveratrol	197	4.23	1.78	−1.85	−3.14

54	Estrone	200	3.03	1.16	−1.97	4.16
55	Simvastatin	198	3.24	3.84	−0.64	1.03
56	Cyclocytidine HCl	38	−3.73	−5.81	1.35	5.97
57	Atropine sulfate monohydrate	200	−5.74	−6.93	−0.67	−2.73
58	Amygdalin	199	−6.42	0.52	3.02	0.92
59	Baicalein	200	−6.96	−7.40	−2.78	1.07
60	Cinchonidine	200	−0.54	1.65	2.12	−1.22
61	DL-Carnitine HCl	202	1.29	0.08	−2.84	−2.77
62	1-Deoxynojirimycin	98	−5.20	−5.13	0.82	2.15
63	Hydroxytyrosol Acetate	199	−7.12	0.62	−4.73	−4.54
64	Lawsone	195	0.93	2.11	−6.21	−7.92
65	Harmine	198	4.04	2.92	−2.61	−0.21
66	Lysionotin	197	3.70	4.43	−2.53	−5.81
67	L-Rhamnose monohydrate	198	13.88	16.93	−3.80	−10.04
68	(-)-Arctigenin	199	17.51	19.49	0.15	−11.42
69	Iso-Steviol	198	25.47	−3.10	−5.69	−9.59
70	Baohuoside I	194	−54.67	−52.88	−11.00	−3.58
71	D-Galactose	200	−7.18	−8.52	−19.77	−15.55
72	Vanillyl Alcohol	195	0.48	0.56	−32.83	−33.42
73	Methyl protocatechuate	196	0.33	0.48	−6.20	−19.19
74	Bavachinin	198	−3.56	−2.09	−2.34	−24.84
75	3,4',5-Trimethoxy-trans-stilbene	200	0.35	0.31	−1.89	−10.33
76	Hydroxy Camptothecine	80	−4.94	−3.46	3.97	−5.83
77	Astragalus polyphenols	199	8.37	7.69	−4.94	−4.44
78	Gracillin	113	−15.13	5.27	−6.46	−5.26
79	Ginsenoside Rb1	90	6.31	12.47	8.71	3.41
80	Eleutheroside B	199	−4.11	−5.92	−2.41	−3.90

81	Glucosamine sulfate	14	−9.20	−9.61	−2.09	−1.62
82	Gallic acid trimethyl ether	198	−2.72	−2.86	−33.89	−26.18
83	Rutin trihydrate	100	−3.83	−4.05	2.83	41.32
84	kaempferide	200	−4.94	−3.60	−3.60	−4.14
85	Arteether	198	−33.89	−26.18	4.12	2.55
86	Hederagenin	199	2.83	3.32	7.06	−0.31
87	Bulleyaconi cine A	11	−3.60	−4.14	3.57	−9.11
88	Macranthoidin B	42	4.12	2.55	15.76	16.95
89	(-)-Epicatechin gallate	199	7.06	−0.31	−13.96	16.78
90	Isoquercitrin	198	−35.69	−9.10	1.26	18.99
91	Camphor	197	55.76	16.95	14.64	20.02
92	Methyl EudesMate	199	−13.96	16.78	38.41	35.22
93	Guaiacol	193	1.26	18.99	16.90	18.70
94	Scopoletin	198	14.64	20.02	12.53	25.96
95	Leonurine	199	38.41	35.22	10.08	12.45
96	Betulonic acid	46	16.90	18.70	10.82	11.08
97	4'-Demethylpodophyllotoxin	200	12.53	25.96	3.14	8.26
98	4',7-Dimethoxy-5-Hydroxyflavone	23	10.08	12.45	2.61	7.25
99	Forsythin	187	10.82	11.08	3.55	3.64
100	Madecassoside	103	3.14	8.26	−1.24	4.79
101	Tetrahydropalmatine hydrochloride	31	2.61	7.25	9.75	14.62
102	Dulcitol	33	3.55	3.64	21.41	22.14
103	Methyl 4-hydroxycinnamate	196	−1.24	4.79	0.55	−1.19
104	Protopine	85	9.75	14.62	12.14	9.02
105	Isopsoralen	75	21.41	22.14	20.79	20.00
106	Astragaloside IV	127	1.03	0.90	0.55	−1.19
107	Catalpol	199	11.14	10.26	12.14	9.02

108	Hederacoside C	57	25.47	20.00	20.79	20.00
